# Prevalence, clinical characteristics, and 3-dimensional radiographic analysis of supernumerary teeth in Guangzhou, China: a retrospective study

**DOI:** 10.1186/s12903-023-03032-9

**Published:** 2023-06-02

**Authors:** Lidan He, Guoying Que, Xiaoxia Yang, Siqi Yan, Song Luo

**Affiliations:** Stomatological Hospital, School of Stomatology, Southern Medical University, Guangzhou, Guangdong Province China

**Keywords:** ST, Characteristics, Eruption potential, CBCT, Panoramic

## Abstract

**Objective:**

The aim was to investigate the prevalence and clinical and 3-dimensional (3D) radiographic characteristics of supernumerary teeth (ST) in a paediatric dental population. The factors associated with ST eruption potential were analysed, and the optimal extraction time for nonerupted ST was discussed.

**Methods:**

A retrospective study was performed in a 13,336-participant baseline population aged 3–12 years for whom panoramic radiographs had been obtained in the hospital from 2019 to 2021. The medical records and radiographic data were reviewed to identify patients with ST. Both the demographic variables and ST characteristics were recorded and analysed .

**Results:**

In total, 890 patients with 1,180 ST were screened from the 13,336 baseline population. The ratio of males (679) to females (211) was approximately 3.2:1. Generally, ST occurred singularly and were frequently found in the maxilla (98.1%). A total of 40.8% of ST were erupted, and the 6-year-old age group presented the highest eruption rate (57.8%). The eruption rate of ST was highly negatively correlated with age. A total of 598 patients additionally underwent cone- beam computed tomography (CBCT). According to the CBCT images, the majority of ST were conical, normally oriented, palatally situated, nonerupted and symptomatic. The most common ST-associated complication was failed eruption of adjacent teeth. In addition, symptomatic ST were more common in the 7- to 8- and 9- to 10-year-old age groups. The eruption rate of ST was 25.3% among the patients who had undergone CBCT. A normal orientation and the labial position were significant protective factors for ST eruption, with odds ratios (ORs) of 0.004 (0.000-0.046) and 0.086 (0.007–1.002), respectively. Age and the palatal position were significant risk factors, with ORs of 1.193 (1.065–1.337) and 2.352 (1.377–4.02), respectively.

**Conclusions:**

This study provides a detailed analysis of ST characteristics in 3–12 year old children. Age as well as the position and orientation of ST were reliable predictors of the ST eruption. An age of 6 years old may be the optimal time for extraction of nonerupted ST to maximize the utilization of eruption potential and reduce the incidence of ST-associated complications.

## Introduction

Supernumerary teeth (ST), one of the most common dental anomalies, are defined as teeth or odontogenetic structures that exceed the normal number of 20 primary teeth or 32 permanent teeth [1]. Mesiodentes are the most common type of ST and are located in the maxillary central incisor region [2]. The prevalence of mesiodentes varies between different racial groups, and there is a higher frequency in the Asian population of approximately 3% [3]. People of any age can have ST, while ST are commonly found in children with mixed dentition. In addition, males are more affected than females (ratio of 3:1) [4]. To date, the aetiology of ST remains unclear. Several scientific hypotheses have been put forward to explain the aetiology and development of ST including atavism, dichotomy of the tooth bud, hyperactivity of the dental lamina, and genetic factors [5]. It has been reported that genetics and heredity play a key role in the occurrence of ST, especially in patients with a syndrome or family history [6]. Environmental factors will increase individual genetic susceptibility [7].

It has been reported that the prevalence of ST among permanent and primary dentition is 1.5-3.5% and 0.3-0.8%, respectively [4, 8]. The reported prevalence of ST varies in different studies due to the different sample populations and diagnostic tools used [9]. A meta-analysis indicated that full-mouth radiographic evaluation was critical for supernumerary tooth identification [10]. However, considering medical ethics, radiographic examination should not be used as a conventional tool for supernumerary tooth screening in the general population [11, 12]. Recently, epidemiological studies of ST have utilized hospital populations who have undergone radiographic evaluations [2, 13, 14]. Although these studies were from hospital populations, the incomparability of results could be caused by the use of different radiographic tools. The two major radiographic tools adopted in the current related literature are panoramic radiography (PR) and cone-beam computed tomography (CBCT). PR is a 2-dimensional (2D) radiographic technique that requires a much lower dose than CBCT [15]. However, this traditional 2D imaging modality fails to provide highly accurate information on ST and the spatial relationships between ST and neighbouring structures and is thus sometimes insufficient for optimal treatment planning and surgery risk assessment [14]. According to the current guidelines, CBCT images should only be obtained when a lower-dose radiological examination, such as panoramic imaging, cannot provide adequate information for clinical diagnosis and treatment [16]. Generally, the information provided by PR is sufficient for some erupted ST, and CBCT images are commonly applied to determine the 3D information of nonerupted ST [14]. The baseline population in many recently published epidemiological reports on ST generally consists of patients who underwent only CBCT imaging [13, 14]. It is supposed that the supernumerary tooth eruption rate assessed by CBCT is lower than the true level [13].

For the treatment of erupted ST, it is generally recommended that ST should be extracted as soon as possible to prevent associated complications, including impacted or delayed eruption, median diastema, displacement or rotation, cyst formation, and root resorption of the adjacent teeth [17–19]. However, the timing of nonerupted supernumerary tooth extraction remains controversial with respect to permanent tooth germ development and potential injury risks, especially for children with mixed dentition [3]. Based on the 25% eruption rate of ST in the literature [14], we supposed that the extraction of some nonerupted ST with eruption potential in appropriate cases can be delayed until self-eruption, and that minimally invasive treatment can be performed for these ST. Therefore, the evaluation of supernumerary tooth self-eruption potential is crucial. The eruption rate of ST has been extensively investigated in many studies [9, 20, 21]. Nevertheless, rather limited attention has been given to the study of the factors associated with supernumerary tooth eruption. Very few related studies have been undertaken to research these factors among 3- to 12-year-old children in Guangzhou, a southern central city of China.

The purpose of this study was twofold, as follows: (i) to present the clinical and 3D characteristics of nonsyndromic ST in Chinese children aged 3–12 years based on medical records and radiographs; and (ii) to analyse the factors related to the supernumerary tooth eruption status based on CBCT images and discuss the optimal extraction time for nonerupted ST.

## Materials and methods

### Study participants

This study consisted of 13,336 patients (7,232 males and 6,104 females) ranging in age from 3 to 12 years who underwent PR at their initial visits to the Affiliated Stomatological Hospital, Southern Medical University, from May 2017 to May 2019. All medical records and radiographs were screened and examined in detail by two professional dentists (Drs. Lidan He and Xiaoxia Yang) independently, and a final consensus was achieved after consultation with another examiner (Dr. Guoying Que) when different diagnoses were reported. Patients who had a history of tooth extraction, maxillofacial anomalies such as cleft lip and palate, or supernumerary tooth-related syndromes such as cleidocranial dysostosis and Gardner’s syndrome were excluded. Patients with at least one supernumerary tooth were enrolled in the current study. Some patients had undergone CBCT additionally after PR. Only the patients who had no tooth extraction history before PR or CBCT were included in the study. Among 13,336 patients with qualified PR data and detailed medical records available, at least one supernumerary tooth was found in 890 patients. Thus, these 890 eligible patients with 1180 ST were enrolled and defined as the research cohort in this study. Of the 890 eligible patients, 598 patients with 825 ST who additionally underwent CBCT were defined as another research cohort.

### Data collection

**Fig. 1:**
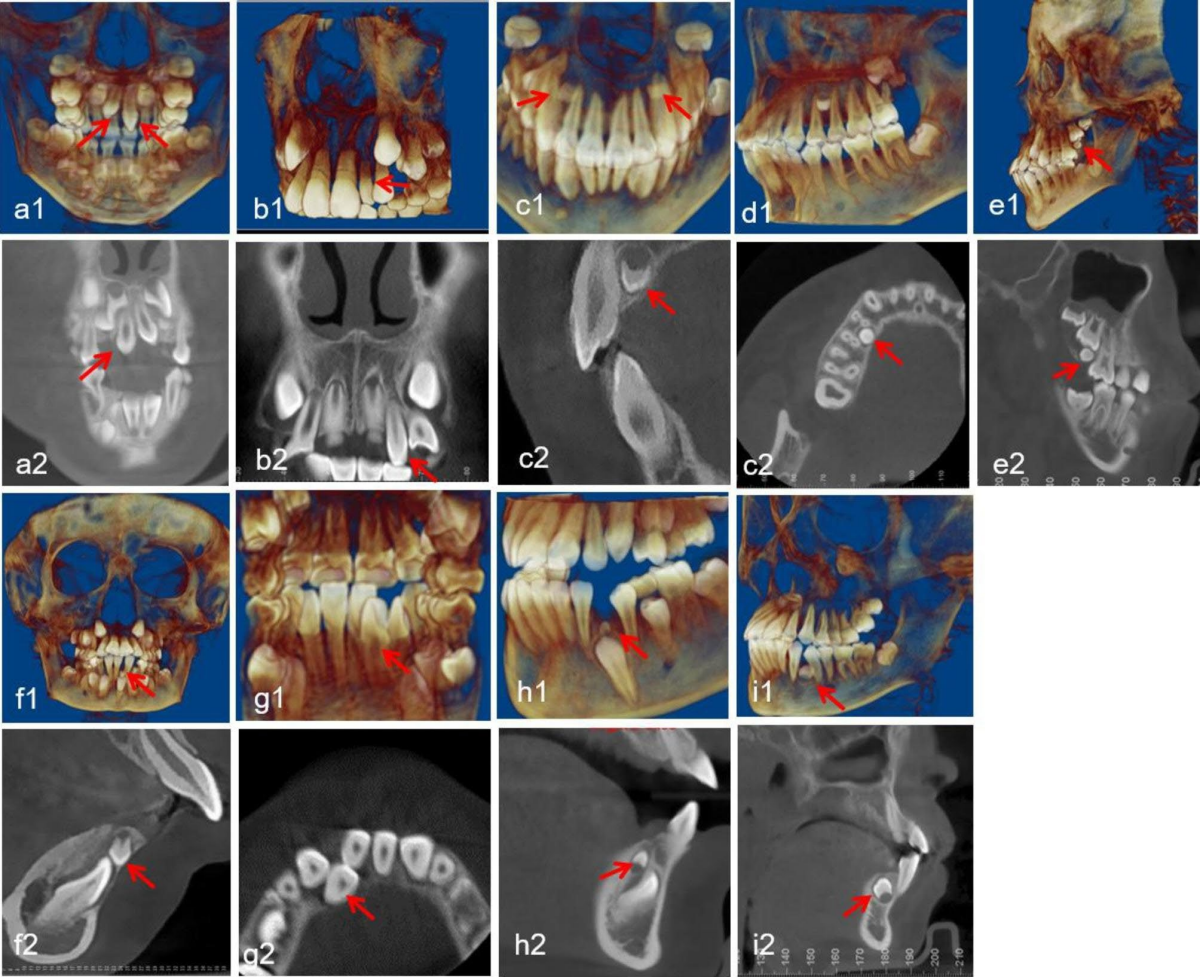
Representative CBCT images exhibiting diverse regions of ST in maxillary and mandibular dental arches. Maxillary arch: (**a1-a2**) central incisor; (**b1-b2**) lateral incisor; (**c1-c2**) canine; (**d1-d2**) premolar; (**e1-e2**) molar. Mandibular arch: (**f1-f2**) central incisor; (**g1-g2**) lateral incisor; (**h1-h2**) canine; (**i1-i2**) premolar. The red arrows point to the ST in the CBCT images

**Fig. 2:**
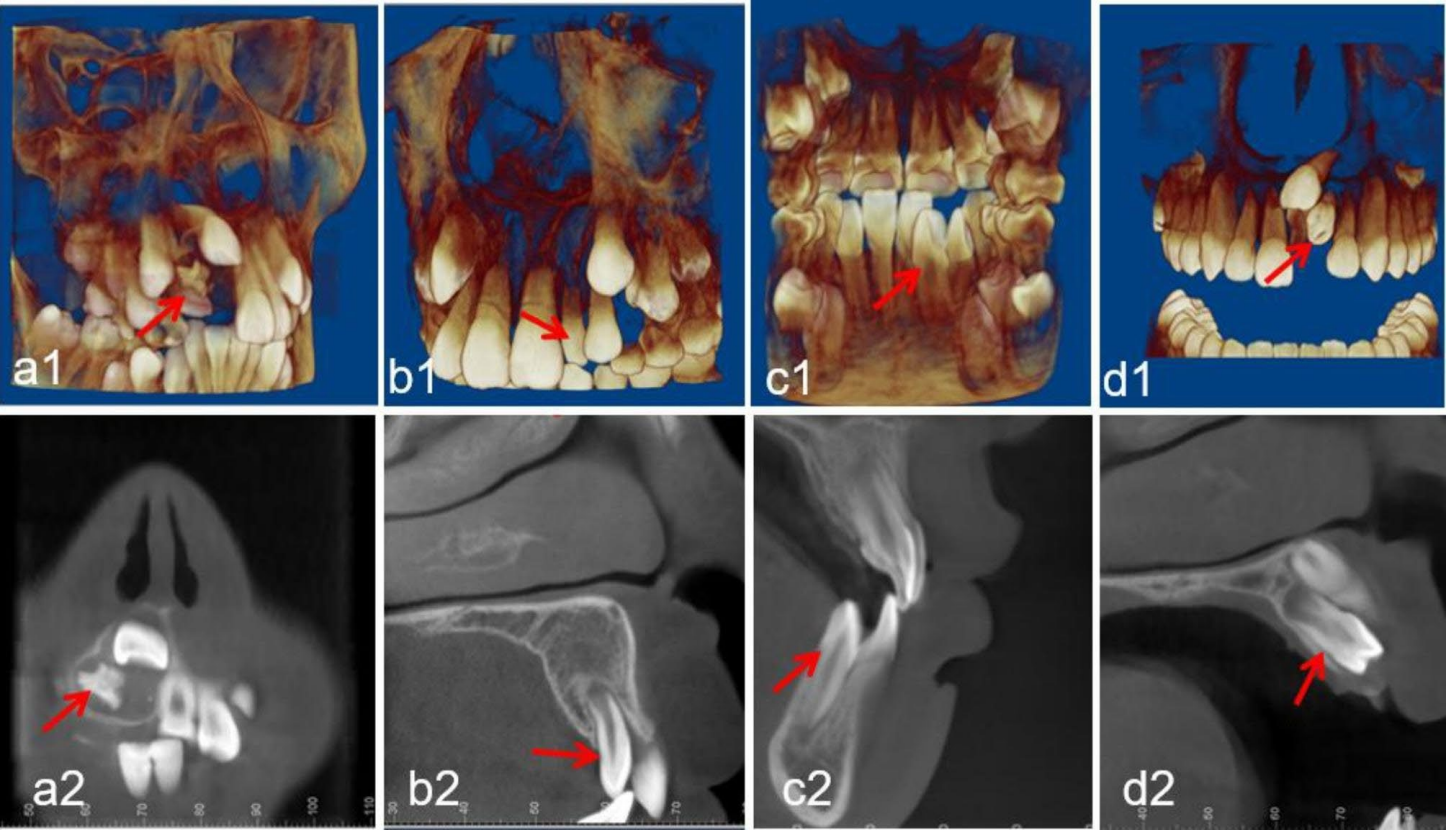
Representative CBCT images exhibiting diverse morphology of ST. (**a1-a2**) odontoid morphology; (**b1-b2**) conical morphology; (**c1-c2**) supplemental morphology of lateral incisor; (**d1-d2**) tuberculate morphology. The red arrows point to the ST in the CBCT images

**Fig. 3:**
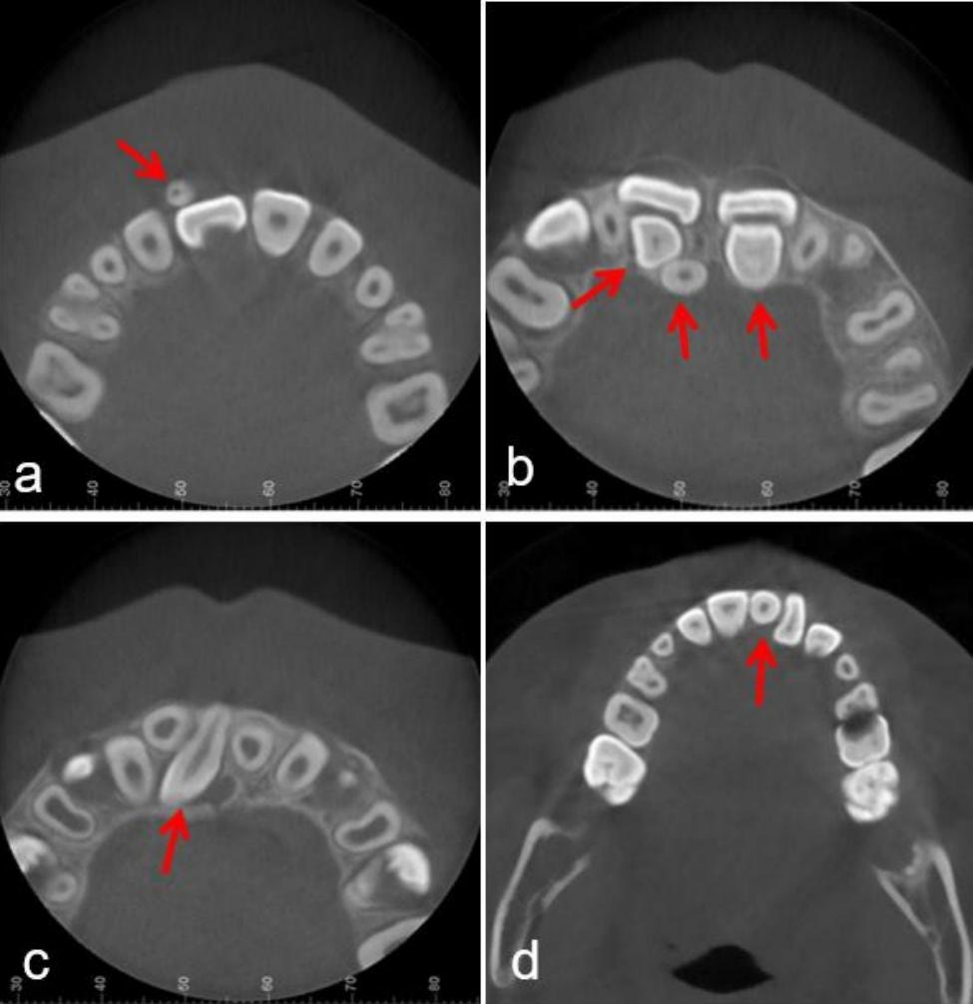
Representative CBCT images exhibiting diverse position of ST. (**a**) labial of the arch; (**b**) palatal of the arch; (**c**) throughout the arch; (**d**) median of the arch. The red arrows point to the ST in the CBCT images

**Fig. 4:**
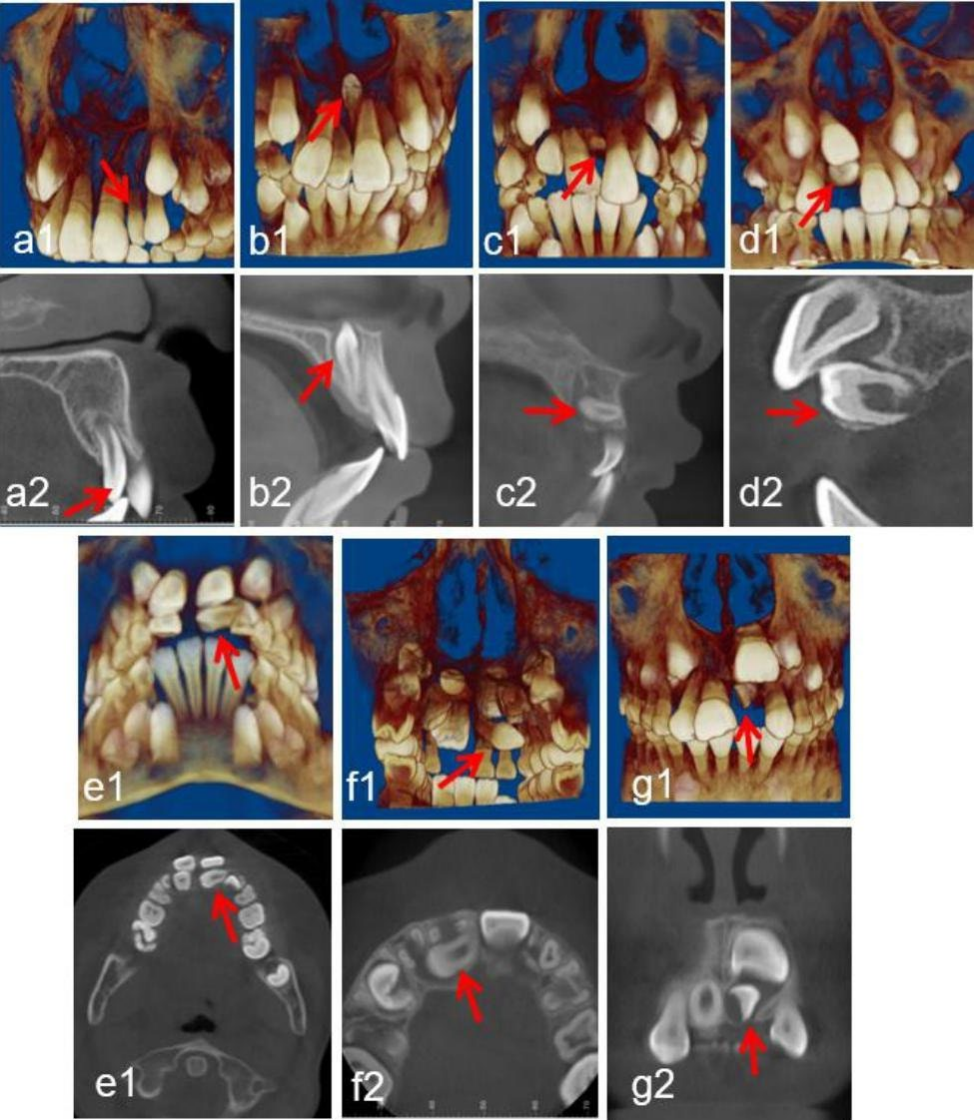
Representative CBCT images exhibiting diverse orientation of ST. (**a1-a2**) normal; (**b1-b2**) inverted; (**c1-c2**) palatal transverse; (**d1-d2**) labial transverse; (**e1-e2**) mesial horizontal; (**f1-f2**) distal horizontal; (**g1-g2**) undefined. The red arrows point to the ST in the CBCT images

**Fig. 5:**
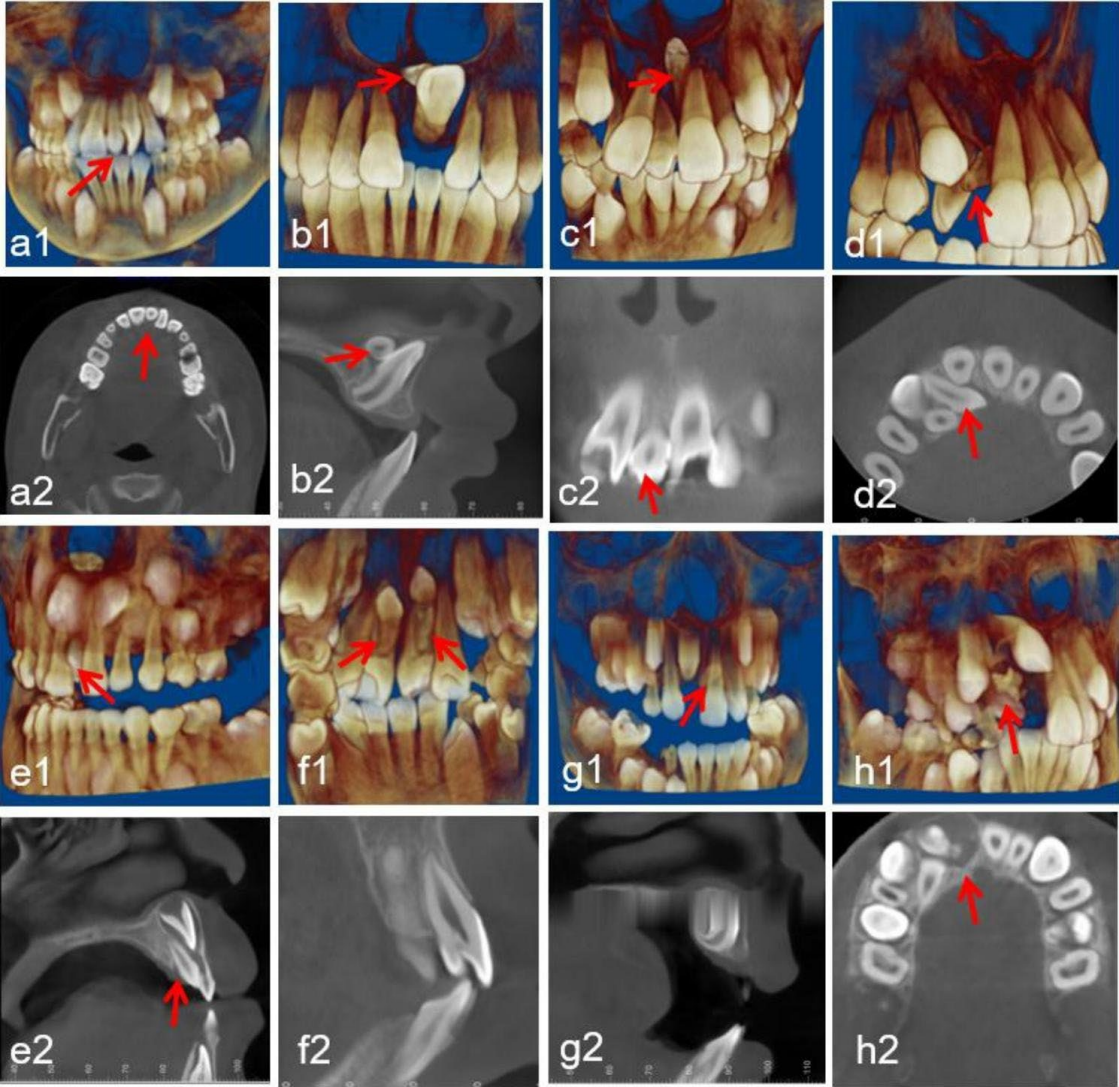
Representative CBCT images exhibiting diverse ST-associated complications of adjacent teeth. (**a1-a2**) rotation; (**b1-b2**) impacted eruption and delayed development; (**c1-c2**) median diastema; (**d1-d2**) displacement; (**e1-e2**) root resorption; (**f1-f2**) enamel invagination; (**g1-g2**) curved root; (**h1-h2**) cyst formation. The red arrows point to the ST in the CBCT images

For the 890 patients with PR, the demographic variables (age and sex) and clinical information (the number and eruption status) of the ST were recorded according to the medical records and radiographic images. Additionally, for patients who underwent both PR and CBCT imaging, we recorded the 3D characteristics (region, morphology, position, orientation, associated complications) of the ST based on the CBCT data coupled with 3D image reconstruction. All CBCT images were captured by a CBCT scanner (3D Accuitomo, Morita Mfg, Corp, Japan) at 85 kV and 5.5 mA. Panoramic radiographs were obtained by a dental panoramic X-ray system (ORTHOPHOS XG PLUS, DS CEPH, Sirona, Germany) at 64 kV and 7 mA. All related CBCT information in DICOM format for every individual patient was collected and imported into Dolphin imaging software (version 11.95, Dolphin, USA), and 3D image reconstruction was performed. Similar to previous reports [13, 17], ST were characterized in detail according to axial, sagittal and coronal CBCT views as well as 3D image reconstruction, as shown below:

1. Region of ST: central incisor, lateral incisor, canine, premolar, and molar (Fig. 1).
2. Morphology of ST: conical, tuberculate, supplemental and odontoid (Fig. 2).
3. Position of ST: buccal/labial, median, throughout the arch and palatal/lingual (Fig. 3).
4. Orientation of ST: normal, inverted, undefined, palatal or labial transverse and mesial or distal horizontal (based on the orientation of the tooth crown) (Fig. 4).
5. ST-associated complications of adjacent teeth: delayed/impacted eruption, displacement, rotation, median diastema, cyst formation, curved root, delayed development, root resorption and enamel invagination (Fig. 5).

### Statistical analyses

All relevant data including the demographic, clinical, and radiographic data of patients were collected and analysed statistically. The interobserver variability and reproducibility of these radiographic measurements were assessed with Cohen’s Kappa coefficient and had values over 0.87. Categorical variables are presented as frequencies and percentages. Associations between the eruption status and 3D characteristics of ST were assessed by Chi-square tests or Fisher’s exact test, as appropriate. Spearman correlation was used to analyse the relationship between the supernumerary tooth eruption rate and age. The Mann-Whitney test was used to analyse the differences in the number distribution of ST in patients of different sexes. Binary logistic regression was used to assess the risk and protective factors for supernumerary tooth eruption. All tests were two-sided, and statistical significance was set with a *P* value of ≤ 0.05. IBM SPSS software (version 24.0; IBM Corp, Armonk, NY, USA) was applied to process the data.

## Results

### Epidemiological characteristics of 890 patients with 1,180 ST based on medical records and panoramic films

**Fig. 6:**
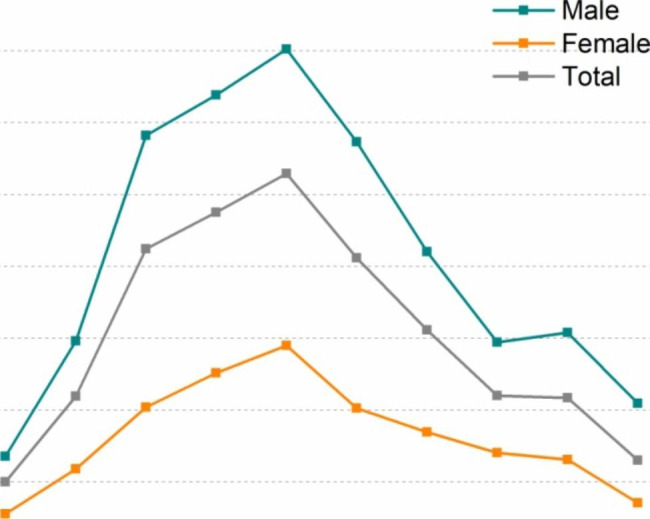
Prevalence of ST in different age groups

In the sample of 13,336 children, 890 patients with at least one supernumerary tooth were identified, for an overall prevalence of 6.67%. Among the 890 patients, there were 679 male (76.29%) and 211 female (23.71%) patients, for a sex ratio of 3.2:1. The ages of the patients ranged from 3 to 12 years, with an average age of 7.7 years. ST were most common in the age range of 5–8 years (Table 1). The prevalence rate among the male and female patients showed a similar tendency with increasing age. The prevalence rate increased first, reached a maximum value at approximately 7 years, and then gradually declined with increasing age (Fig. 6).

**Fig. 7:**
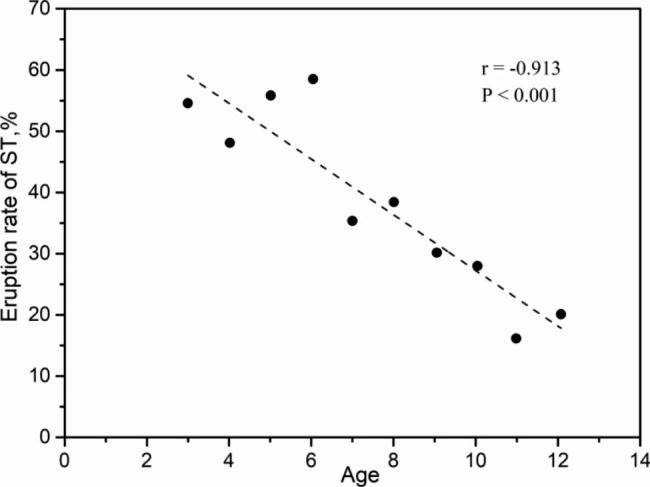
Eruption rate of ST in different age groups

A total of 1,180 ST were found in the 890 patients, and the average number of ST per patient was 1.33. There was no significant difference in the number of ST between patients with primary and mixed dentition (*P* > 0.05). Most patients had a single supernumerary tooth (68.2%), followed by patients with two ST (31.2%), while more than two ST were found in less than 1.0% of patients (Table 2). The percentage of patients with ST in the maxilla (98.1%) was greater than that of patients with ST in the mandible (1.6%). Very few (0.3%) patients had ST in both the maxilla and mandible. According to the clinical records and panoramic images of 890 patients, 482 (40.85%) ST were erupted. Six-year-old patients showed the highest eruption rate (57.80%). The eruption rate of ST was strongly negatively correlated with age (r = -0.913, *P* < 0.001) (Fig. 7).

**Table 1:** Prevalence and male/female ratio of ST in the 13,336-participant baseline population based on different age groups

Age	n/N (%)^a^						Male/Female^b^
	Male		Female		Total		
3	6/221	(2.71)	2/180	(1.11)	8/401	(2.00)	2.4:1
4	30/506	(5.93)	9/382	(2.36)	39/888	(4.39)	2.5:1
5	88/756	(11.64)	22/539	(4.08)	110/1295	(8.49)	2.9:1
6	125/979	(12.77)	36/716	(5.03)	161/1695	(9.50)	2.5:1
7	147/1047	(14.04)	44/758	(5.80)	191/1805	(10.58)	2.4:1
8	110/960	(11.46)	30/740	(4.05)	140/1700	(8.24)	2.8:1
9	68/809	(8.41)	21/619	(3.39)	89/1428	(6.23)	2.5:1
10	38/645	(5.89)	17/605	(2.81)	55/1250	(4.40)	2.1:1
11	38/617	(6.16)	17/649	(2.62)	55/1266	(4.34)	2.4:1
12	29/692	(4.19)	13/916	(1.42)	42/1608	(2.61)	3.0:1
Total	679/7232	(9.39)	211/6104	(3.46)	890/13,336	(6.67)	2.7:1

^a^ n: number of patients with ST; N: number of patients as the baseline population, (%): prevalence of ST.

^b^ Male/female: prevalence ratio of supernumerary tooth in males relative to females.

**Table 2:** Number distribution of ST in male and female patients

Sex	One n (%)	Two n (%)	Three n (%)	Four n (%)	Z	*P* value
Male	450 (66.3)	225 (33.1)	3 (0.4)	1 (0.1)	-2.209	0.027*
Female	157 (74.4)	53 (25.1)	0 (0.0)	1 (0.5)		
Total	607 (68.2)	278 (31.2)	3 (0.3)	2 (0.2)		

*Statistical significance (*P* ≤ 0.05). The *P* value was calculated by the Mann-Whitney test using SPSS 24.0.

**Table 3:** Morphology distribution of 825 ST within different regions based on CBCT images

Region		Morphologies of ST, n (%)				
		Conical	Tuberculate	Supplemental	Odontoid	Total
Maxilla	Central	563(74.8)	157(20.8)	15(2.0)	18(2.4)	753
	Lateral	16(57.1)	7(25.0)	4(14.3)	1(3.6)	28
	Canine	8(50.0)	5(31.3)	0(0.0)	3(18.8)	16
	Premolar	1(14.3)	0(0.0)	4(57.1)	2(28.6)	7
	Molar	1(50.0)	0(0.0)	0(0.0)	1(50.0)	2
	Total	589	169	23	25	806
Mandible	Central	1(100.0)	0(0.0)	0(0.0)	0(0.0)	1
	Lateral	0(0.0)	0(0.0)	1(100.0)	0(0.0)	1
	Canine	2(66.7)	0(0.0)	0(0.0)	1(33.3)	3
	Premolar	3(21.4)	2(14.3)	6(42.9)	3(21.4)	14
	Total	6	2	7	4	19

### Three-dimensional characteristic analysis of 825 ST based on 598 CBCT images

A total of 598 patients underwent both PR and CBCT, and their ST were analysed by using 3D reconstruction. A total of 825 ST were found in the 598 patients. The numbers of ST located in the maxilla and mandible were 806 and 19, respectively.

### Region and morphology distribution

Regarding the region of ST, ST located in the maxillary central incisor area had the highest proportion, accounting for 91.3% (753 teeth), followed by ST in the maxillary lateral incisor region (28 teeth), maxillary canine region (16 teeth), and mandibular premolar region (14 teeth). In the present paper, the morphologies of ST were classified as conical, tuberculate, supplemental and odontoid [9]. Conical was the most common type (72.1%), followed by tuberculate (20.7%), supplemental (3.7%), and odontoid (3.5%). The detailed region and morphology distributions of the ST in the maxillary and mandibular dental arches are shown in Table 3.

### Position and orientation distribution

Regarding the position of 825 ST relative to the dental arch, the palatal/lingual region (534 teeth) was the most common position, followed by the middle of the dental arch (155 teeth), throughout the arch (122 teeth), and the buccal/labial position (14 teeth). The orientations of the ST were classified into seven subtypes according to the CBCT images: normal, inverted, undefinable, palatal or labial transverse and mesial or distal horizontal (based on the orientation of the supernumerary tooth crown). A normal orientation was the most common (320 teeth), followed by inverted (275 teeth), palatal transverse (138 teeth), undefined (61 teeth), labial transverse (16 teeth), mesial horizontal (10 teeth) and distal horizontal (5 teeth).

### ST-associated complications

Several complications in adjacent teeth affected by ST have been reported, such as delayed/impacted eruption, displacement, rotation, cyst formation, and root resorption of the neighbouring tooth.

**Fig. 8:**
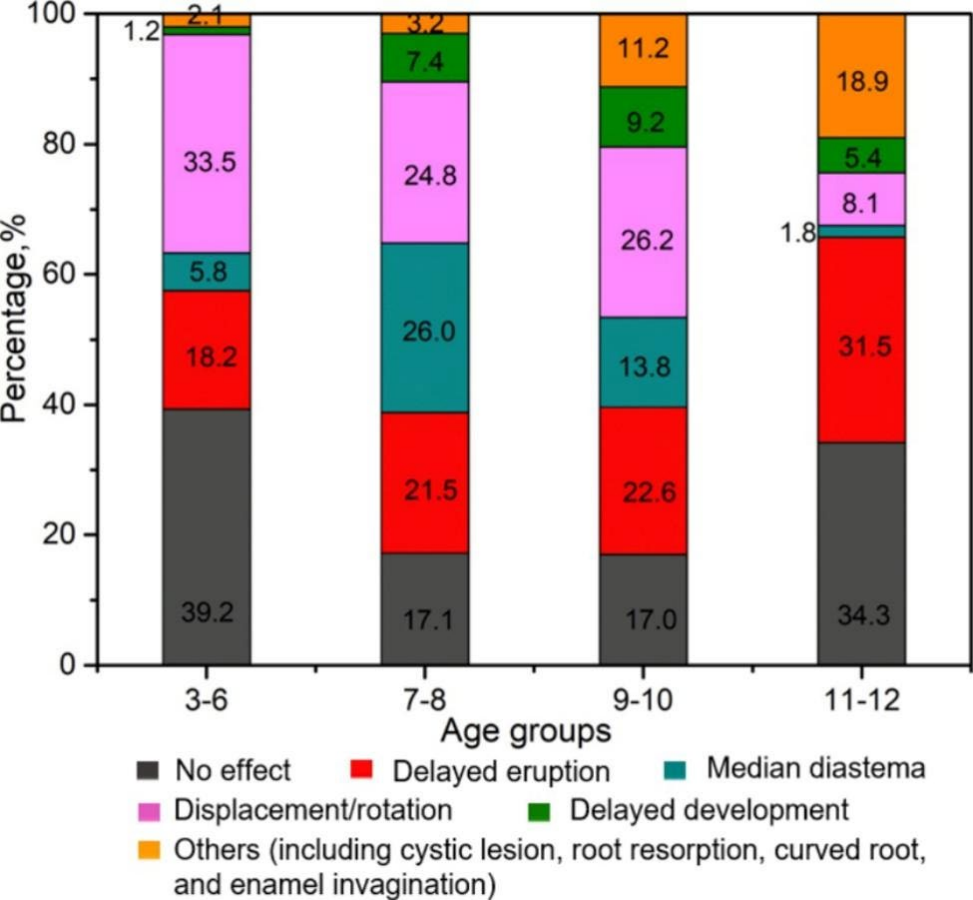
Bar chart showing the complication distribution of ST in different age groups

In this work, 825 ST were found on CBCT images, 589 (71.4%) of which were associated with complications. Some ST were associated with more than one complication. Among all ST-related complications, failed eruption of the adjacent teeth was the most common (211, 35.8%), followed by median diastema (149, 25.3%), rotation (138, 23.4%), and displacement (100, 17.0%). In addition, other less common complications in adjacent teeth were observed, such as delayed development, enamel invagination, cystic formation, root resorption, and curved root formation. As shown in Fig. 8, more ST presented without complications in the 3- to 6- and 11- to 12-year-old age groups than in the 7- to 8- and 9- to 10-year-old age groups.

### Factors associated with the status of 825 ST with 3D reconstruction

In total, of the 825 ST, 209 ST (25.3%) were erupted, and 616 ST were nonerupted. Based on the clinical records and CBCT images, the associations of age, sex, and supernumerary tooth morphology, region, position, and orientation with eruption status were analysed (Table 4). A statistically significant difference was found for patient age (*t*=-7.336, *P* < 0.05). The average age of patients with erupted ST was 6.88 years (± 1.81), which was lower than that of patients with nonerupted ST (7.98 years ± 2.06). Analysis of the data in Table 4 indicates that the region, morphology, orientation, and position of ST could have statistically significant effects on the eruption of ST (*P* < 0.05).

### Binary logistic regression analysis of the factors related to supernumerary tooth eruption

The supernumerary tooth eruption status was taken as the dependent variable (0 = eruption, 1 = noneruption). The variables with *P* ≤ 0.05 in the univariate analysis were included in the binary logistic regression model. In the final model, three independent variables were entered into the equation (Table 5): age, orientation, and position. Regarding protective factors, ST in a normal orientation had a 99.6% lower chance (OR = 0.004, 95% CI = 0.000-0.046, *P* < 0.001) of noneruption than ST in other orientations. ST in a labial position had a 91.4% lower chance (OR = 0.086, 95% CI = 0.007–1.002, *P* = 0.05) of noneruption than ST in the median of the dental arch. Regarding risk factors, older age was significantly associated with noneruption compared with younger age (OR = 1.193, 95% CI = 1.065–1.337, *P* = 0.002). Similarly, a palatal position was significantly associated with noneruption compared with a median position (OR = 2.352, 95% CI = 1.377–4.02, *P* = 0.002).

**Table 4:** Factors related to the supernumerary tooth eruption status regarding diverse 3D characteristics

Studied factors		Status				χ^2^	*P* value
		Nonerupted (n,%)		Erupted(n,%)			
Region	Central incisor	554	73.5%	200	26.5%	10.291	0.027*
	Lateral incisor	22	75.9%	7	24.1%		
	Canine	18	94.7%	1	5.3%		
	Premolar	20	95.2%	1	4.8%		
	Molar	2	100.0%	0	0.0%		
Morphology	Conical	449	75.5%	146	24.5%	15.274	0.002*
	Tuberculate	120	70.2%	51	29.8%		
	Supplemental	18	60.0%	12	40.0%		
	Odontoid	29	100.0%	0	0.0%		
Orientation	Normal	118	36.9%	202	63.1%	440.655	< 0.001*
	Inverted	275	100.0%	0	0.0%		
	Palatal transverse	133	96.4%	5	3.6%		
	Labial transverse	15	93.7%	1	6.3%		
	Others^a^	75	98.7%	1	1.3%		
Position	Palatal	418	78.3%	116	21.7%	82.537	< 0.001*
	Throughout	114	93.4%	8	6.6%		
	Median	76	49.0%	79	51.0%		
	Labial	8	57.1%	6	42.9%		

*Statistical significance (P ≤ 0.05). The *P* value was calculated by the chi-square test or Fisher’s exact test using SPSS 24.0.

^a^ Others include mesial or distal horizontal and undefinable orientations.

**Table 5:** Results of logistic regression analysis of the eruption status of ST with related factors

Studied factors	B	S.E.	Wald	df	*P* value	OR	95% CI for OR	
							Lower	Upper
Age	0.177	0.058	9.244	1	= 0.002*	1.193	1.065	1.337
Orientation			38.807	4	*<*0.001*			
Normal	-5.583	1.281	18.983	1	*<*0.001*	0.004	0.000	0.046
Inverted	15.954	2383.093	0.000	1	0.995	8490272.592	0.000	
Palatal transverse	-1.146	1.369	0.7	1	0.403	0.318	0.022	4.656
Labial transverse	-1.511	1.62	0.87	1	0.351	0.221	0.009	5.28
Position			15.805	3	0.001			
Palatal	0.855	0.273	9.791	1	= 0.002*	2.352	1.377	4.02
Labial	-2.449	1.251	3.834	1	0.05*	0.086	0.007	1.002
Throughout	-0.207	0.873	0.056	1	0.812	0.813	0.147	4.499
Constant	3.201	1.369	5.467	1	0.019	24.553		

OR = odds ratio; CI = confidence interval; * Statistically significant (*P* ≤ 0.05).

^a^ Others include mesial or distal horizontal and undefinable orientations.

## Discussion

The precise causes of ST are unclear. Several hypotheses have been proposed in previous studies, such as atavism, dichotomy of the tooth bud, hyperactivity of the dental lamina, and genetic factors [22]. It is believed that genetic factors are an important cause of ST [23–25]. McBeain et al. found that 20.5% of patients with ST had first-generation relatives who also had ST [15]. The prevalence of ST in the Caucasian population is lower than that in the Mongolian population [3]. Asian countries or regions such as India and Hong Kong have a higher prevalence of ST (1.40-2.97%) [26–28], and the prevalence of ST in Australia and Rome is generally lower (0.28-0.66%) [15, 28, 29]. The present study covered 13,336 participants in a baseline population and revealed a 6.67% prevalence rate of ST among the total population. The prevalence rates among males and females were 9.39% and 3.46%, respectively, which are greater than those in previous studies [2, 13, 14]. One reason for this difference could be that most subjects have mixed dentition. Previous studies have indicated that the prevalence of ST among children with mixed dentition is higher than that among subjects in other age groups [30, 31].

Because panoramic films are usually used for initial supernumerary tooth screening [10], patients who have undergone panoramic imaging in dental hospitals were selected as our surveyed subjects. A Hong Kong study reported that the supernumerary tooth prevalence rate among 1,093 randomly sampled 12-year-old students in school undergoing PR was 2.7% [28]. The prevalence rate of ST among 12-year-old patients in the present study was similar (2.61%, 42/1608).

The supernumerary tooth prevalence rate increased first, reached a maximum value at approximately 7 years, and then gradually declined with increasing age. For patients aged 3–7 years, the supernumerary tooth prevalence rate increased gradually with age, which could be due to the following reasons. On the one hand, ST gradually begin to erupt or cause related complications with increasing age, thereby increasing the demand for medical treatment. On the other hand, the development time and location of supernumerary tooth germs are uncertain, and late-developing ST will appear with age [32].

Patients aged 7 years had the highest prevalence rate, which may be due to the onset of maxillary incisor eruption at this age and a number of ST-related complications, such as delayed eruption of neighbouring teeth. Among patients aged 7–12 years, the supernumerary tooth prevalence rate decreased slowly with age. The reason may be that erupted ST or ST with complications were extracted in the early dentition stage. The remaining ST were mainly without complications or buried in the bone and were incidentally discovered by radiological examination.

Noticeably, there was no significant difference in the prevalence of ST between the primary dentition (6.1%, 157/2584) and mixed dentition (6.8%, 733/10,752) populations (P > 0.05). In this study, the prevalence of ST in the primary dentition population was far more than the 0.3 − 0.8% previously reported in the literature [4]. The difference is mainly attributed to the diagnostic methods. Previous studies on ST in primary dentition mainly used visual diagnosis, resulting in a large number of missed diagnosis [11, 12].

The occurrence of ST was related to sex, and males were diagnosed more often than females. This result is consistent with the findings in the literature [15, 33, 34]. In the present work, the prevalence of ST among males relative to females was 2.7:1. Recently, many studies on the genes and family epidemiology related to ST have been published [24, 35]. Autosomal or sex chromosome heredity has been proposed to be an important aetiological factor [36]. Our results further supplement the epidemiological data of ST.

In the present study, the number of ST ranged from 1 to 4. A single supernumerary tooth (68.2%) was the most common, followed by 2 ST (31.2%), 3 ST (0.3%) and 4 ST (0.2%). Multiple ST (≥ 5 ST) are very rare and are usually associated with a syndrome, such as cleidocranial dysplasia [6]. A similar study was reported by Bereket, and the results suggested that 77.4% of patients had one supernumerary tooth, 18.4% of patients had 2 ST, and 4.2% of patients had 3 or more ST [37].

The location distribution of ST was different in the upper and lower jaws. The location of maxillary ST was basically symmetrical. ST were distributed mainly near the central incisor area, followed by the lateral incisor area, canine area, premolar area, and molar area. Mandibular ST were most often found in the premolar area, followed by the canine and incisor areas, while ST were not detected in the molar area. It is worth noting that a rare case of ST in the mandibular central incisor region was observed, as presented in Fig. 1. It is usually believed that ST rarely occur in the mandibular central incisor area [38].

It has been reported that 47.6-88.5% of ST cause complications in patients [38, 39]. Our results show that 71.4% of ST had related complications. Notably, the manifestations of the ST-associated complications were different in each age group. Considering that most ST were located in the maxillary incisor area and that the eruption period of the maxillary incisors is approximately 7–9 years of age [4], it can be concluded that most cases of ST in the 7- to 8- and 9- to 10-year age groups included related complications owing to the effect of ST on the eruption of maxillary incisors. In the 7- to 8-year age group, the main complication of ST was median diastema, followed by displacement/rotation and failed eruption of adjacent teeth. The most common complication in the 9- to 10-year age group was displacement/rotation of adjacent teeth, followed by abnormal eruption and median diastema. Delayed/impacted eruption of adjacent teeth was the most common complication in the 11- to 12-year age group, followed by other rare complications (Fig. 5). It is suggested that the later the intervention is performed, the more difficult the subsequent treatment will be [3].

Previous studies have reported that approximately 1/4 of ST can erupt [14, 40]. According to the PR and medical records of the 890 patients, 482 of the 1,180 ST erupted, for a rate of 40.9%, which is higher than the rate previously reported in the literature [40]. However, among the 598 patients who underwent CBCT, the supernumerary tooth eruption rate was 25.3%, which is consistent with the eruption rate of 25.0% that was previously reported in the literature [13, 14]. The reason for the difference in the eruption rate could be related to the inconsistency of the diagnostic tools. In general, CBCT is mostly used for nonerupted ST in the clinic. Some studies [13, 14] only included supernumerary tooth patients who underwent CBCT, which could cause the supernumerary tooth eruption rate to be far lower than the actual rate.

Generally, while erupted ST can be extracted, extraction is not always suitable for nonerupted ST, especially in children with permanent tooth germ development in their jaws [41]. Currently, the best timing for the extraction of nonerupted ST in this population is still controversial. Alsani et al. suggested that the early extraction of ST (before 6 years) can effectively prevent the related complications of ST and can maximize the eruption potential of permanent teeth [42]. It is emphasized that ST should be removed as soon as they are found. However, considering that the developing permanent tooth germ could be damaged during the operation, some authors advocate delaying extraction until the patient is 8 to 10 years old, when the roots of the adjacent teeth are basically developed [3]. However, the disadvantage is that delayed extraction could cause adjacent teeth to lose their eruption potential. Then, secondary surgery and/or orthodontic interventions could be needed in later stages [43].

The present study showed that six-year-old patients had the highest eruption rate (57.80%) and that the eruption rate of ST was strongly negatively correlated with age. Meanwhile, after the age of six, ST-associated complications were more likely to occur. Therefore, the age of 6 years old may be a suitable time for the extraction of nonerupted ST. The results of this study also showed that the 3D characteristics (orientation and position) of ST were associated with the eruption status of the ST, suggesting that the CBCT evaluation of nonerupted ST for accurate planning is recommended. However, the cross-sectional design of this study had some limitations, and longitudinal studies should be conducted in the future to further confirm the results.

## Conclusion

The study provides a detailed analysis of the characteristics of ST in 3–12 year old children in Guangzhou, China. The prevalence of ST was 6.7%, and ST were more common in male patients. The prevalence of ST was the highest in the 7-year-old group (10.58%), and the eruption rate was the highest in the 6-year-old group (57.80%). The majority of the ST were conical, normally oriented, palatally situated, nonerupted and symptomatic. Age as well as the position and orientation of ST are reliable predictors of the supernumerary tooth eruption status. Six years of age may be a suitable time for the extraction of nonerupted ST to maximize utilization of the eruption potential and reduce the incidence of ST-associated complications. Due to the limitations of cross-sectional studies, longitudinal studies should be conducted in the future to further confirm the results.

## Data Availability

The data that support the results of this study are available from the corresponding author upon reasonable request.

## Abbreviations

2D: 2-dimensional
3D: 3-dimensional
CBCT: Cone- beam computed tomography
ORs: Odds ratios
PR: Panoramic radiography.
ST: Supernumerary teeth
